# Expression of calgranulin A/B heterodimer after acute inhalation of endotoxin: proteomic approach and validation

**DOI:** 10.1186/1471-2466-13-65

**Published:** 2013-11-15

**Authors:** Olivier Michel, Virginie Doyen, Baptiste Leroy, Benjamin Bopp, Duc Huy Phong Dinh, Francis Corazza, Ruddy Wattiez

**Affiliations:** 1 Clinic of Immuno-allergology, CHU Brugmann (ULB), pl Van Gehuchten 4, B-1020 Brussels, Belgium; 2 Hematology, CHU Brugmann (Université Libre de Bruxelles - ULB), pl Van Gehuchten, 4, B-1020 Brussels, Belgium; 3 Department of Proteomics and Microbiology, University of Mons, Mons, Belgium; 4 Microarray Unit, Jules Bordet Institute (ULB), Brussels, Belgium; 5 Department of Immunology, Pham Ngoc Thach University of Medicine (PNTU), Ho Chi Minh City, Vietnam

**Keywords:** Endotoxin, Inflammation, Sputum, Proteomic, Calgranulin, Neutrophils

## Abstract

**Background:**

The acute inhalation of endotoxin mimicks several aspects of the inflammation related to chronic obstructive pulmonary disease (COPD). The aim of the current study was to identify and to validate biomarkers of endotoxin-induced airways’ inflammation.

**Methods:**

The cellular count in the induced-sputum, was measured before and after an inhalation of 20 mcg endotoxin, in 8 healthy volunteers. A proteomic analysis was applied to identify the more relevant proteins expression, before measurement by ELISA. The amplitude and the repeatability of the markers were evaluated among another population of 12 healthy subjects.

**Results:**

There was a significant rise of viable cells (p <0.01), macrophages (p <0.05), and neutrophils (p <0.02) 24 hours after endotoxin inhalation, and of neutrophils (p <0.02) and lymphocytes (p <0.05) at 6 hours. Among the highest amplitude responses, the two dimensional electrophoretic separation shown proteolytic activity and overexpression of protein spots. By MALDI-TOF mass spectrometry, the last were identified as calgranulin A and B. The expression of the bioactive A/B heterodimeric complex was confirmed by ELISA both in the sputum (p <0.01) and at the blood level (p <0.01). The intra-subject repeatability of the sputum calgranulin A/B was highly significant (p <0.0001).

**Conclusion:**

In healthy subjects, the inhalation of endotoxin induced expression of sputum calgranulin A/B that could be a biomarker of the endotoxin response/exposure.

## Background

Endotoxin and its purified derivative lipopolysaccharide (LPS) are pro-inflammatory constituents from Gram-negative bacteria, present in a variety of occupational and home environments [1] and in cigarette smoke [2]. In the airways, LPS is signalling through the Toll-like receptor-4 (%TLR4), expressed by the stromal cells of the lung [3].

In healthy subjects, an acute inhalation of LPS produces fever and flu-like symptoms, a rise of sputum polymorphonuclear neutrophils (sPMN) and inflammatory mediators, a blood increase and activation of neutrophils and an increase of the C-reactive protein (CRP) [4-6]. It was proposed that this inflammatory response could be a model to evaluate anti-inflammatory drugs [7-10]. However the limitation is the large inter- and intra-subject variation of the amplitude of the response [11,12], due to both genetic factors and methods to measure the inflammatory response [13]. One important factor of variation is the saliva contamination during the plugs selection. To improve the strength of the LPS model, there is a need for a valid bronchial inflammatory marker, in regard with the neutrophilc activation.

In the present study, larger responders to LPS inhalation were selected among a group of healthy subjects. Instead of measurement of a number of markers of cells activation, a proteomic analysis was applied to identify possible markers of the lung injury among the selected subjects and to evaluate the saliva contamination. Then, the highlighted biomarkers were measured by ELISA among all the subjects. Afterwards, the repeatability of the biomarker was evaluated in both sputum and serum.

## Methods

### Subjects

Population A. Nine healthy non smoker volunteers of age 18–50 were able to produce an adequate induced-sputum (defined as viability of > 70%, squamous cells < 50% and a percentage of PMN < 50%). The reason to select subjects with a low basal inflammation (< 50% PMN) was to increase the chance to observe a larger inflammatory response to LPS, before proteomic analysis. One subject having declined to participate to the LPS challenge, eight subjects (subjects 1 to 8) were included (36.7 (± 2.4) years; F/M = 4/4). The study was approved, by the Ethical Committee (decision number 04-03-7/2617 of the National Register) of the Institution (CHU St-Pierre).

Population B. A second population of 12 healthy non smoker volunteers (subjects 9 to 20) was included (37.2 (±2.3) years; F/M = 9/3). They were able to produce an adequate induced-sputum without limits of the % of PMN. The study was approved by the Ethical Committee (decision CE2010/09 13-01-2010) of the Institution (CHU Brugmann).

Informed written consent was obtained in each subject from both populations.

### General design

On day 0, a sputum was induced (defined as basal sputum) among the subjects of population A. On days 14 and 28, each subject was exposed to LPS by inhalation. An induced-sputum was sampled at 6 or 24 hours, in random order, after each LPS exposure. By doing so, we avoided an interference of saline [14] and repeated LPS inhalations [15] on the response to LPS. The procedure of LPS challenge has been previously reported [4,12]. Briefly 20 μg of a suspension of LPS (Escherichia coli 026:B6 from Sigma Chemical, St Louis, MO -ref L-2654) was administered by a Mefar dosimeter MB3 (Mefar, Brescia, Italy). Symptoms, oral temperature, forced vital capacity (FVC), forced expiratory volume in 1 second (FEV1) and the FEV1/FVC were recorded before and hourly after LPS.

In the 2d part of the study, the population B was challenged with inhaled LPS. The blood was sampled before, 6 and 24 hours after LPS, while the sputum were induced 7 days before and 24 hours after the LPS challenge. To evaluate the reproducibility of the response, the LPS challenges were repeated after a 2 weeks of wash-out. This period is enough, since we have shown previously that the LPS induced sputum inflammation normalized after 7 days [15]. The mean of each parameter was calculated.

### Induced sputum

Hypertonic sterile saline (5%) was nebulized for 30 minutes with an ultrasonic nebulizer (Fisoneb; Karapharm, Marseille, France); subjects rinsed their mouth with water every 10 minutes and tried to cough sputum directly into a sterile plastic box. After selection of all portions of sputum as free as possible of saliva [16], the plugs were weighed, mixed with 4 volumes of dithiotreitol 0.1% (Sputolysin; Behring Diagnostics, Somerville, NJ), homogenised and rocked for 15 min. before adding 4 volumes of Dulbecco’s PBS. After filtration and centrifugation (15 minutes at 800 g) the supernatant was frozen at -80°C while the pelleted cells were resuspended in PBS. The number of total cells was measured with a Thoma’s hemocytometer. The cell viability was assessed by the Trypan blue method. A slide was prepared by centrifugation (Cytospin, Shandon Inc, Pittsburgh, PA) and stained with May-Grünwald-Giemsa. The percentage of the cells were counted on a total of 400 cells.

### Proteomic analysis of the sputum

Prior to two dimensional electrophoretic separation, supernatants of sputa were extensively dialysed against rehydratation buffer (8 M urea, 2% (w/v) CHAPS, pharmalyte 2% (v/v) and 50 mM DTT). 50 μg of proteins were then subjected to isoelectric focusing on immobilized pH gradient (IPG) strips (pH 3–10; NL; 18 cm; Amersham Pharmacia Biotech, Sweden). The first-dimension isoelectric focusing was carried out as previously described [17]. For the second dimension, thin precast horizontal gels (ExcelGel XL SDS 12–14, Amersham Biosciences) were run for three hours at 40 mA. Gels were stained with a mass spectrometry compatible silver staining procedure as described previously [17].

### Proteins identification

The silver-stained protein spots of interest (taking into account the intensity and repeatability of the spots) were cut out and distained by incubation of gel pieces in 200 μl of 0.003 mM K_4_[Fe(CN)_6_], 0.01 mM Na_2_S_2_O_3_ for 15 minutes. Gel pieces were then washed twice in 25 mM NH_3_HCO_3_ for 15 minutes under gentle agitation at room temperature, followed by two 15 minutes washes in 25 mM NH_3_HCO_3_, 50% (v/v) CH3CN. After Speed vacuum dehydration, 10 μl of a 0.02 μg/μl trypsin solution in 25 mM NH_3_HCO_3_ (Promega Madison), were added, and samples were incubated overnight at 37°C. Tryptic digestion was stopped by addition of 1 μl of 5% (v/v) formic acid. For MALDI-TOF analysis, 1 μl of each sample was mixed with 1 μl of matrix (5 mg/ml æ-cyano-4-hydroxycinnamic acid and 0.5 pmol/μl rennin as internal standard, in 25% (v/v) ethanol, 25% (v/v) acetonitrile, 0.05% (v/v) TFA), then spotted onto a MALDI sample plate and allowed to air dry. MALDI-TOF was performed using a M@ldi™ mass spectrometer (Micromass, Manchester, UK) equipped with a 337 nm nitrogen laser. The instrument was operated in the positive reflectron mode with 15 kV of source voltage, 2.5 kV of pulse voltage and 2 kV of reflecting voltage. Proteins identification was realized using ProteinLynx global server (Micromass) in Swiss-prot (release 54.2) and TREmBL (release 37.0) database without species restriction, using mass tolerance of 100 ppm, carbamidomethylation of Cysteine as fixed modification and oxidation of methionine as variable modification.

### Calgranulin assays in sputa and sera

Due to the presence of large amount of reducing agent, sputa were extensively dialysed against 50 mM Tris–HCl pH 7.4 at 4°C before calgranulin assay by ELISA. Calgranulin A and B and calgranulin A/B complex, were assayed in sputa (from population A) using the calgranulin A and B assay kits from DPC (USA), and the calgranulin assay kit from BMA biochemical (CH), respectively, following manufacturer instructions. Calgranulin A/B complex was assayed in sera and sputa of population B, using the calgranulin assay kit from BMA biochemical (CH, product code: S-1011). The lower limit of sensitivity of the assay is 20 ng/mL. Sputa were tested undiluted.

### Interferon-gamma (IFN), Interleukin-6 (IL-6) and interleukin-8 (IL-8) assays in sputa

The concentrations of IL-6, IL-8 and IFN in the supernatants of the sputa were measured by flow cytometry, using the Cytometric Bead Array Human Inflammatory Kit from BD Biociences (San Jose, CA), with a theoretical limit of detection 2.5 pg/mL for IL-6 and 3.6 pg/mL for IL-8.

### Statistics

The data were expressed as means ± SE, after log transformation when required. Means were compared by paired or non-paired t-tests. Repeatability was assessed by plotting the differences between repeated measurements against the mean of the repeated measures, and testing whether the mean differences was significantly different from 0 (method of Bland and Altman) [18]. Associations were assessed by linear regression analysis. A p value < 0.05 was considered as significant.

## Results

**Figure 1:**
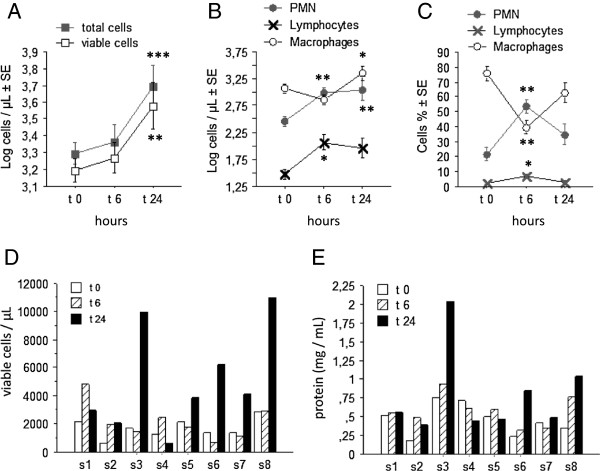
**The sputum cells response induced by LPS. A**: The mean values of the total and viable cell count. **B** and **C**: the mean changes of the cells differential (expressed in absolute **(B)** and relative **(C)** values) before, 6 and 24 hours after LPS inhalation. D/E: The individual changes of the total viable cell count **(D)** and of the concentration of proteins **(E)** from the sputum, before, 6, 24 hours and 7 days after LPS inhalation. Results are expressed as means (±SE). Statistics: paired t-tests. * p > 0.05; ** p < 0.02; *** p < 0.01.

The response to inhaled endotoxin in population A:

Except subject 4 who complained of slight fatigue and headache, there was no significant symptom, and on response of lung function or fever 24 hours after LPS. At the sputum level, the rise of log PMN was significant 6 and 24 hours after LPS, while the lymphocytes rose at 6 hours and the macrophages at 24 hours (Figure 1).

The significant rise of the total protein concentrations (p < 0.02) and of the total viable cells (p < 0.02) into the sputum, 24 hours after LPS, were characterized by variable amplitude between the subjects (Figure 1). The three subjects displaying the larger response (subjects 3, 6, 8) were selected to evaluate the protein content by 2DE approach.

**Figure 2:**
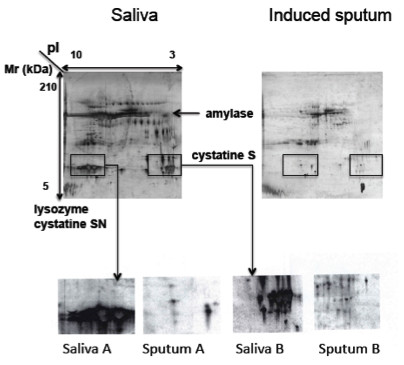
**The 2DE analysis of sputum compared to saliva.** The major salivary proteins, Cystatine S, Amylase and Lysozyme, were observed on saliva gel and clearly absent in the sputum.

A two dimensional electrophoretic separation was applied on the sputum supernatants. The contamination of the sputum samples by saliva was excluded because major salivary proteins, Cystatine S, Amylase and Lysozyme, were observed on saliva gel and clearly absent in the sputum (Figure 2). The reproducibility of sputa analysis by 2DE was tested by running three times the same sample and analyzing, by use of PDQuest, the resulting gels in terms of spot numbers and relative intensity for 40 randomly selected spot. There was no significant difference between these triplicates (data not shown). The inter-individual variability has also been tested. In this context, three sputa samples, at the basal state, from different subjects were analysed by 2DE. While the protein profile and number of spots seemed very similar, the relative intensities of 40 randomly selected spots showed significant differences. Nevertheless, this inter-individual variability was in the range of what is generally observed for other biological fluids.

**Figure 3:**
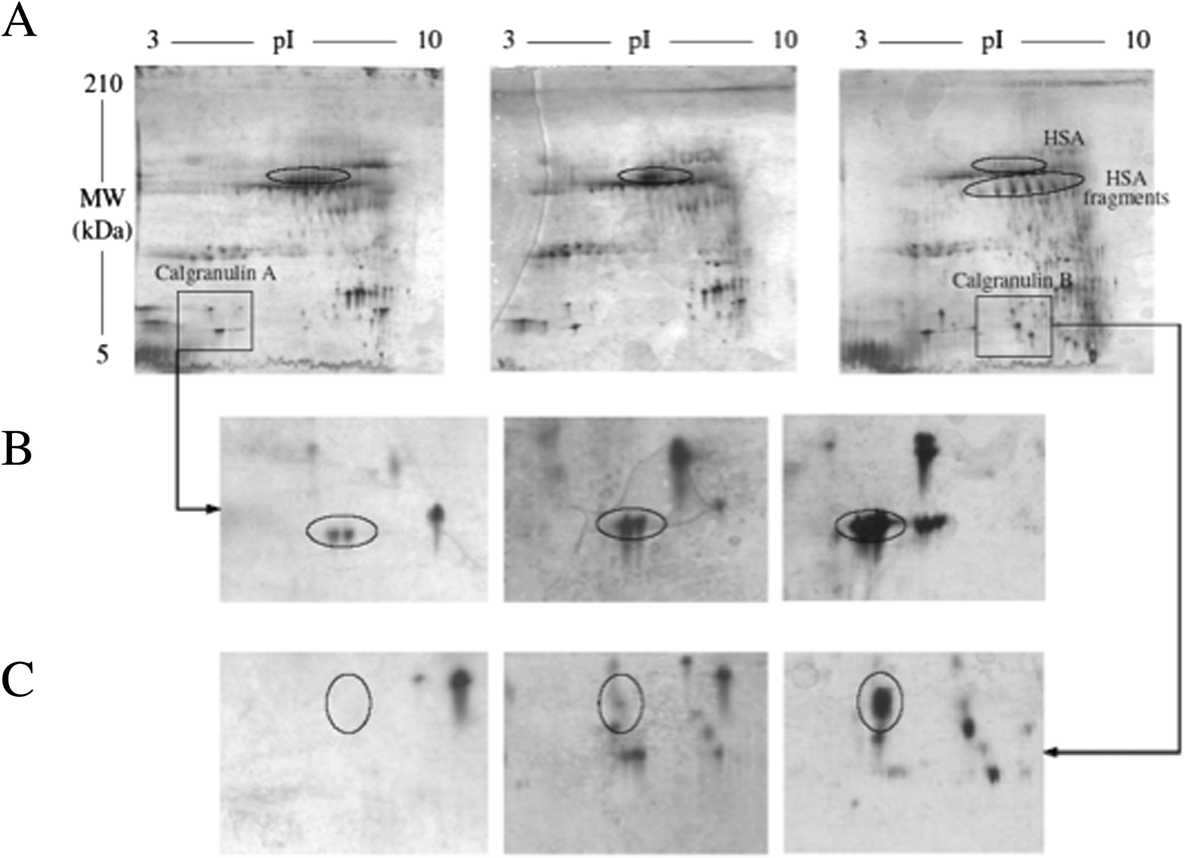
**The 2DE analysis of LPS induced sputa.** Samples collected after different recovery time (0, 6 and 24 hours) after LPS inhalation were analyzed by 2DE and compared. This analysis clearly shows HSA degradation **(A)** and a rise in calgranulin A and B abundance **(B and C)**.

Compared to time 0, at times 6 and 24 hours after PLS, a decrease in intensities of protein spots of human serum albumin (HSA) was observed, in association with the expression of a number of protein spots corresponding to HSA fragments (shown by decrease of MW in SDS page), suggesting the occurrence of a proteolytic activity (Figure 3). A significant increase in two others protein spots was highlighted (Figure 3). Mass spectrometry analysis identified these protein spots as calgranulin A and B.

**Figure 4:**
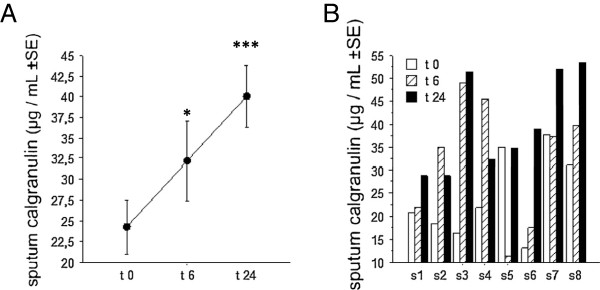
**The sputum calgranulin response induced by LPS. A**: the mean (±SE) concentrations of calgranulin A/B complex (ELISA) in sputum before, 6 and 24 hours after endotoxin inhalation. **B**: the individual values of the calgranulin A/B heterodimeric complex concentration in sputum before, 6 and 24 hours after endotoxin inhalation. Statistics: paired t-tests. * p < 0.05, *** p < 0.01.

Then, the calgranulin A and B concentrations were measured by ELISA in the sputa of all subjects of the population. We observed an increase (though not significant) of calgranulin A but not of calgranulin B, 24 hours after LPS. In fact, the calgranulin A and B are secreted as a heterodimeric complex which are not well assayed by the ELISA for the calgranulin monomers. Consistently, calgranulin A/B heterodimeric complex testing revealed a significant increase in the sputum supernatant, 6 (p < 0.05) and 24 hours (p < 0.01) after LPS (Figure 4).

### The response to inhaled endotoxin in population B

**Figure 5:**
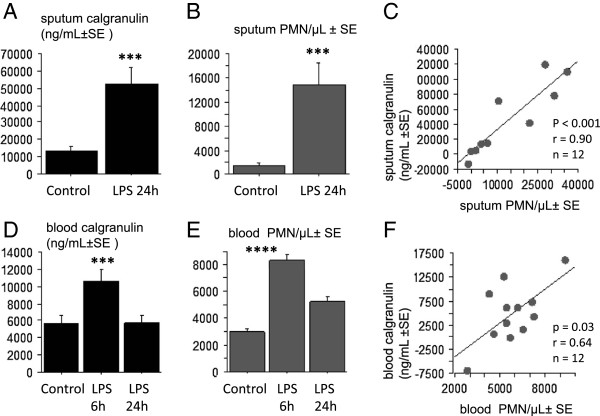
**The relationship between the calgranulin and neutrophilic responses induced by LPS.** The LPS-induced inflammatory response in the 2d population. **A**: the significant increase of sputum calgranulin A/B complex, 24 hours after endotoxin inhalation. **B**: the significant rise of the neutrophils (PMN) in the sputum, 24 hours after endotoxin inhalation. **C**: the linear correlation between the rises in sputum calgranulin and sputum PMN, at 24 hours after LPS inhalation, compared to basal value. **D**: the significant increase of blood calgranulin A/B complex, 6 hours after endotoxin inhalation. **E**: the significant increase of blood neutrophils, 6 hours after endotoxin inhalation. **F**: the linear correlation between the rises in blood calgranulin and blood PMN, 6 hours after LPS inhalation, compared to basal value. Statistics: paired t-tests. *** p < 0.01, **** p < 0.001.

The mean squamous cells was 66.7 (± 21.1) cells/μL corresponding to 6.9 (±1.9)% of the total cells, suggesting a low saliva contamination, according to the proteomic analysis in population A. A significant neutrophilic response with an expression of calgranulin A/B complex was confirmed 24 hours after LPS in the sputum (Figure 5A-B). There was also a rise of neutrophils and calgranulin A/B complex after 6 hours at the blood level, that normalized at 24 hours (Figure 5D-E). At the basal state, the sputum concentration of calgranulin A/B was not correlated with the PMN in sputum. Conversely, the LPS-induced rises of sputum calgranulin correlated significantly (p < 0.0001) with the rise of PMN (Figure 5C). At the blood level the calgranulin A/B expression correlated (but less significantly, p < 0.03) with the rise of neutrophils (Figure 5F).

**Figure 6:**
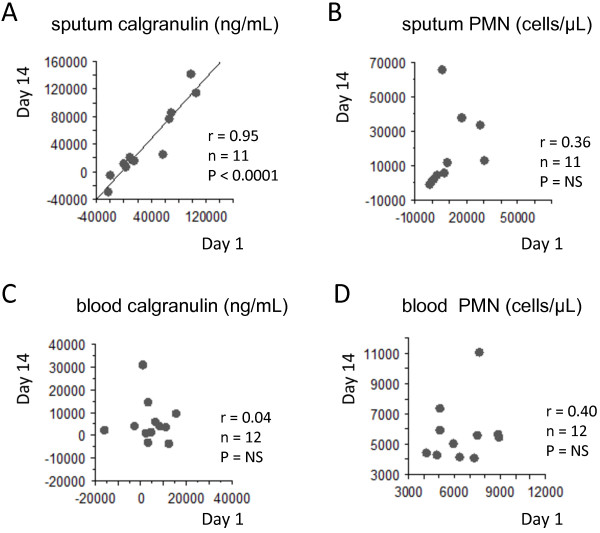
**Assessment of repeatability of the sputum/blood calgranulin A/B and neutrophils, induced by inhaled LPS.** The linear significant correlation between post-LPS sputum calgranulin A/B repeated at 2 weeks interval, among 11 subjects **(A)**. The absence of significant correlation between repeated (at 2 weeks interval) post-LPS neutrophils **(B)**, blood calgranulin **(C)** and blood neutrophilia **(D)**.

The challenges were repeated at 2 weeks interval. The Bland and Altman analysis (the differences against the means, between the day 1 and 7) showed significant repeatability both for the sputum calgranulin and neutrophilic responses. While there was a significant linear correlation between post-LPS sputum calgranulin A/B repeated at 2 weeks intervals, it was not significant for the sputum post-LPS neutrophils (Figure 6). In fact, the figure shown that the repeatability of PMN post-LPS was related with the amplitude of the PMN response, larger responses being less repeatable (Figure 6). The repeatability of both the neutrophilic and the calgranulin responses were not significant at the blood level (Figure 6).

The cytokines Il-6 and IL-8 increased significantly after LPS inhalation (from 8.2 ± 2.2 to 104.7 ± 36.1 pg/mL, p < 0.02 and 800.4 ± 175.9 to 2416.2 ± 782.9 pg/mL, p < 0.05, respectively). When the LPS inhalation was repeated at two weeks interval, the correlation was significant for each repeated marker (r = 0.79 p < 0.01 and r = 0.61, p < 0.05 for IL6 and IL8, respectively). Compared to calgranulin, the sputum concentrations of IL6, IL8 and PMN count were less repeatable after LPS inhalation at 2 weeks interval (Table 1).

**Table 1:** Linear correlation of sputum biomarkers after repeated LPS inhalation at 2 weeks interval

**Sputum biomarkers**	**n**	**F-test**	**r**	**p**
Calgranulin	12	77.0	0.95	< 0.0001
IL-6	12	16.3	0.79	0.0024
IL-8	12	5.7	0.61	0.0372
PMN (%)	10	4.1	0.58	0.0774
PMN (absolute value)	10	1.2	0.36	0.3051
Macrophages (absolute value)	10	0.2	0.16	0.6557

## Discussion

An inhalation of endotoxin induced expression of the calgranulin A/B heterodimeric complex, at the sputum and blood levels. The rise of the sputum calgranulin A/B complex and PMN were significantly repeatable and related, in a given subject.

Instead of measuring several inflammatory parameters, we chose the proteomic approach to identify the end-points, after having selected the subjects characterized by a larger amplitude response. Indeed, the present study confirmed that the changes in the cells count and the protein concentration induced by an endotoxin inhalation, is highly variable between subjects [8,9], this individual variability being associated with several gene polymorphisms [19,20].

The proteomic analysis on bronchoalveolar lavage fluid (BALF) has been used successfully for the detection of biomarkers in COPD or cystic fibrosis [21,22]. However sampling of BALF requires bronchoscopy, an invasive procedure that cannot be performed in all patients and/or repeated in healthy subjects. Though, the technique of induced sputum can be repeated at different time-points in a given subject. Available data have provided a first “catalogue” of 191 sputum proteins obtained by sputum-induction from healthy subjects [23]; the present data confirm that the intra- and inter-individual repeatability of 2DE was suitable for sputum proteome analysis and that the contamination by saliva was not significant.

In response to endotoxin inhalation, two major phenomena can be distinguished at the proteome level. Firstly, an important proteolytic activity was suggested by the disappearance of whole albumin and by the increase in the amount and the number of albumin fragments on the 2D map. Consistently, in airways inflammatory diseases, the proteomic analysis identified extensive protein degradation such as in cystic fibrosis (sputum) [22], or in smokers with COPD (BALF) [18]. Future studies will investigate the clinical relevance of this observation. The second endotoxin induced phenomenon is the overexpression of calgranulin A and B. These proteins, also called protein S100 or MRP (myeloid-related protein), are calcium-binding proteins involved in many regulatory activities such as the dynamic of cytoskeleton constituents, cell growth and differentiation, and calcium homeostasis [24]. These two proteins were assayed in sputa after LPS inhalation, but only confusing variation were observed in the present study. In fact, calgranulin A and B are secreted as the tetramer formation of S100A8/A9 [24] having biological activity [25]. Our current data showed a significant rise in abundance both at the blood and sputum levels of the calgranulin heterodimeric A/B complex. Consistently, Gray et al. reported that sputum proteomics, in inflammatory and suppurating respiratory diseases, identified biomarkers such as ELISA-measured calgranulin (heterodimer of calgranulins A and B), which is clinically relevant and associated with the treatment [26]. Wittkowski et al. have shown that in 8 healthy subjects (compared to subjects exposed to saline), S100A12 was elevated in bronchoalveolar lavage fluid 6 hours after LPS inhalation [27]; we extended these data, showing that the response was measurable both in the induced-sputum and blood and it was larger at 24 hours, compared to 6 hours.

The induced-sputum technique allowed repeating samples in a given subject. By doing so, we can demonstrated that the amplitude of the endotoxin-induced rise of PMN and calgranulin A/B complex were significantly reproducible in a given subject. Though, the LPS-induced PMN, IL-6 and IL-8 responses were less repeatable. The calgranulin A/B complex rise from the non apoptotic phagocytes is characterized by a larger amplitude (increase more than 5 times) and by an immediate diffusion into the mucus, that could explain a better repeatability, compared to the neutrophils.

In the present study, others soluble markers in the sputum, such as IL-6 and IL-8 were reproducible after repeated LPS inhalation, though with a lower significance compared to the calgranulin. In regard with the neutrophilic characteristics of the LPS induced inflammation, this is probably due to the source of IL-6 and IL-8 related to others cells than neutrophils such as monocytes and lymphocytes. It is only recently, that the question of repeatability of the LPS response has been investigated. R Kitz et al. have published a first study with repeated LPS challenges in human volunteers [7]. At the systemic level they reported a good repeatability for the blood inflammatory proteins (CRP) and neutrophilia, though there was a tendancy of rising the amplitude of the response, in some subjects, after the 4th repeated exposure. Except eNO (that did not change significantly), there was no measurement of marker of bronchial inflammation. In a more recent paper, O. Janssen et al. reported a good reproducibility of sputum % of PMN at 6 hours after an inhalation of a low dose (2 μg) LPS [28]. Our results shown a less reproducibility of % of PMN (r = 0.58, n = 10), that could be due to the sampling time at 24 hours (instead of 6 hours), when the sputum % neutrophilia is decreasing (see Figure 1 of the present study). They also measured MPO concentration in sputum that was repeatable for the population as a whole [28]. While the MPO and neutrophil elastase were not measured in the present study, it would be an interesting comparator in future validation experiments.

Innate immunity plays a primary role in infectious and non-infectious diseases, which is initiated by the recognition of microbial determinants, such as LPS [26]. In concert with these pro-inflammatory substances, endogenous molecules released by activated or damaged cells, are able to activate the innate immunity mechanisms. This group of molecules is called “endokines”, “alarmins” or “damage-associated molecular patterns (DAMP)”. One example of this substance group is the phagocytes S100 proteins [24]. The DAMP are sensed by the toll-like receptors (TLRs) and the receptor for advanced glycation end products (RAGE) [29]. The S100 proteins are calcium binding proteins and include more than 20 different proteins. The S100A8 (calgranulin A or MRP8) and S100A9 (calgranulin B or MRP14), are found in cells of myeloid origin [30]. This is supported by the current data showing a relationship between the LPS-induced rises in calgranulin and PMN. The S100A8 and A9 proteins, up-regulate the adhesion receptor of phagocytes [31], bind to endothelial cells [30], and increases the vascular permeability [32]. Vogl et al. have demonstrated that S100A8 interacts with the TLR4-MD2 complex, which represent an endogenous ligand of TLR4, that can amplify phagocyte activation while antibodies against S100A8 and A9 blocked the LPS-induced phagocytes migration [33]. A role of calgranulin A/B complex in the endotoxin-induced reaction is also supported by clinical studies having related the S100A8/A9 expression with suppuratiing respiratory diseases [26], respiratory distress syndrome [27] and COPD [21].

In the present study, we also showed that the LPS-induced calgranulin A/B heterodimeric complex was significant not only in the sputum but also at the blood level, representing a more accessible sample. However the lack of intra-subject repeatability of this parameter limits its potential interest.

Thus, in healthy subjects, the inhalation of endotoxin induced a relevant and reproducible expression of sputum calgranulin A/B complex. This biomarker could be relevant to evaluate the in vivo responsiveness to inhaled endotoxin in human.

## Conclusion

There is no valide and relevant measurable marker of the inflammatory reponse to endotoxin (a promising model to evaluate future anti-inflammatory drugs in human). By proteomic approach we identified the calgranulin A/B heterodimeric complex in the sputum, and we showed that this response was related with the neutrophilic response, and that the repeatability was higher for calgranulin, compared to the neutrophils. The data are of interest for the researchers developping strategies to evaluate drugs in their early phase of developement, in future studies.

## Abbreviations

BALF: Bronchoalveolar lavage fluid; COPD: Chronic obstructive pulmonary disease; CRP: C-reactive protein; DAMP: Damage associated molecular pattern; ELISA: Enzyme-linked immunosorbent assay; FEV1: Forced expiratory volume in one second; FVC: Forced vital capacity; HSA: Human serum albumin; LPS: Lipopolysaccharide; MALDI-TOF: Matrix-assisted laser desorption/ionisation - time-of-flight mass spectrometry; PBS: Phosphate buffer saline; PMN: Polymorphonuclear neutrophils; TLR: Toll-like receptor; TNF: Tumor necrosis factor.

## Competing interests

The authors have declared that they have no conflict of interests.

## Authors’ contributions

OM: concept of the study, writing of the paper. BP, BB, RW: proteomic investigation, ELISA (part). DHPD and VD: data collection, contribution to the writing of the paper. FC: sputum analysis and ELISA (part). All authors read and approved the final manuscript.

## Pre-publication history

The pre-publication history for this paper can be accessed here:

http://www.biomedcentral.com/1471-2466/13/65/prepub
